# Neural activity in the dorsal medial superior temporal area of monkeys represents retinal error during adaptive motor learning

**DOI:** 10.1038/srep40939

**Published:** 2017-01-19

**Authors:** Aya Takemura, Tomoyo Ofuji, Kenichiro Miura, Kenji Kawano

**Affiliations:** 1 Human Informatics Research Institute, National Institute of Advanced Industrial Science and Technology, Ibaraki 305-8568, Japan; 2 Department of Integrative Brain Science, Graduate School of Medicine, Kyoto University, Kyoto, 606-8501, Japan

## Abstract

To adapt to variable environments, humans regulate their behavior by modulating gains in sensory-to-motor processing. In this study, we measured a simple eye movement, the ocular following response (OFR), in monkeys to study the neuronal basis of adaptive motor learning in the visuomotor processing stream. The medial superior temporal (MST) area of the cerebral cortex is a critical site for contextual gain modulation of the OFR. However, the role of MST neurons in adaptive gain modulation of the OFR remains unknown. We adopted a velocity step-down sequence paradigm that was designed to promote adaptive gain modulation of the OFR to investigate the role of the dorsal MST (MSTd) in adaptive motor learning. In the initial learning stage, we observed a reduction in the OFR but no significant change in the “open-loop” responses for the majority of the MSTd neurons. However, in the late learning stage, some MSTd neurons exhibited significantly enhanced “closed-loop” responses in association with increases in retinal error velocity. These results indicate that the MSTd area primarily encodes visual motion, suggesting that MSTd neurons function upstream of the motor learning site to provide sensory signals to the downstream structures involved in adaptive motor learning.

Everyday life requires that we move accurately and effortlessly within various environments. We achieve this by regulating the magnitude of our movements, even simple sensory-to-motor reflexive actions such as eye movements. The magnitude of the movements that are elicited by sensory stimulation can be considered a “gain” in the system when sensory input and motor output have the same dimension. The regulation of gains in the reflexive system allows adaptive motor learning, which is important for straightforward feedback to the motor control system. Because most eye movements require a direct transformation from sensory input into an appropriate motor command in the absence of immediate sensory feedback, the study of the oculomotor system could be useful in understanding the neural mechanisms that are involved in adaptive motor learning. Therefore, the goal of the current study was to understand the neural basis of adaptive motor learning, which controls the execution of appropriate motor behaviors by regulating the gain in the reflexive system.

The ocular following response (OFR) is a reflexive slow tracking eye movement that is evoked by the sudden movement of a visual scene. The OFR has a short latency (~50 ms in monkeys and ~80 ms in humans) and is thought to help stabilize the position of the eyes on the visual scene when the observer moves^1^,^2^. The gain of the OFR is defined as the ratio of the input velocity (movement of a scene) to the output velocity (eye movement). Previous studies have reported that a gain in OFR can be modulated by several conditions, including the vergence angle (a major viewing distance cue)^3^,^4^, binocular disparity (a depth cue)^5^, preceding saccades (post-saccadic enhancement)^6^, and visually mediated adaptive regulation^7^. The vergence angle and binocular disparity help isolate the images in the plane of fixation when the moving observer looks to one side. Post-saccadic enhancement helps foveate an object when the moving observer inspects a nearby object. Visually mediated adaptive regulation improves the OFR by maintaining the visual stability irrespective of changes in the oculomotor apparatus.

Previous lesion and single-unit recording studies in monkeys have revealed that the OFR is mediated by the medial superior temporal (MST) area in the dorsal stream of the cerebral cortex and by its projection to the ventral paraflocculus (VPFL) of the cerebellum via the dorsolateral pontine nucleus (DLPN)^8^,^9^,^10^,^11^,^12^. Several single-unit recording studies have provided further evidence for the role of the MST area in the regulation of the gain of the OFR^13^,^14^,^15^. First, the magnitude of the OFR is inversely proportional to the viewing distance and is related to the ocular vergence angle^3^,^4^. Inoue, *et al*.^13^ investigated the dependence of the OFR on vergence in monkeys and found that about half of the MST neurons (45%, 72/160) increased their firing rate with the increasing convergence of the eyes^13^. Second, the earliest OFRs are tuned to the image in the immediate vicinity of the plane of fixation^5^. Takemura, *et al*.^14^ studied the effect of binocular disparity on early-OFRs and found that over half of the MST neurons (55%, 41/75) had disparity tuning curves that resembled those of the OFRs^14^; this finding suggested that MST neurons may contain information on binocular disparity. Finally, OFRs showed a transient enhancement after saccadic eye movements, which is termed “post-saccadic enhancement”^6^. Takemura, *et al*.^14^ reported that most MST neurons (83%, 40/48) with OFR-related activity show a similar dependence on the post-saccadic delay^15^,^16^. These studies suggest that the activities of the MST neurons might be causally linked to the regulation of the gain of the OFR. In these cases, the gain of the OFR synchronously changed with the changes in the inner/outer environment, and thus, this type of modulation can be referred to as a “contextual” modulation of the OFR. Repeated exposure to a new environment evokes gradual adaptive changes of motor behavior to sensory stimuli in a process known as “adaptive motor learning” or “adaptive gain modulation”. However, the role of the MST area in “adaptive” gain modulation of the OFR during motor learning has not been studied. Although there is a prominent theory of cerebellar motor learning^17^,^18^, it is unknown whether the MST area similarly functions in both adaptive gain modulation and contextual gain modulation.

In this paper, the adaptive gain modulation of OFR during motor learning was investigated by adopting a previously proposed adaptation paradigm: velocity step-down sequences. The paradigm was designed to initiate an OFR at the beginning and then to induce consistent visual errors (retinal errors) by decreasing the stimulus velocity (velocity step-down) after the OFR initiation. OFRs were expected to be effectively reduced by repeated exposure to the velocity step-down sequences. We hypothesized that if the MST area was the site of or at a site downstream of the adaptive gain modulation, neuronal activity would change in parallel with OFR modulation. Conversely, if neuronal activity did not change in parallel with OFR modulation, these conclusions would not be accepted. Toward these goals, we recorded single unit activities of neurons in the MST area of the monkey cortex.

## Results

We recorded the responses of 58 direction-selective neurons from the dorsal part of the MST (MSTd) to the adaptation stimulus in five hemispheres of three awake behaving monkeys (see Supplementary Table S1). Repeated exposure to velocity step-down movements (a decrease to 0°/s in the second step) of the visual scene resulted in considerable decreases in the OFRs. In the adaptive gain modulation, these decreases can be considered adaptive because they operate to reduce the net retinal error during the second step (150–300 ms from the stimulus onset) of the adaptation paradigm^7^.

### Behavior

**Figure 1:**
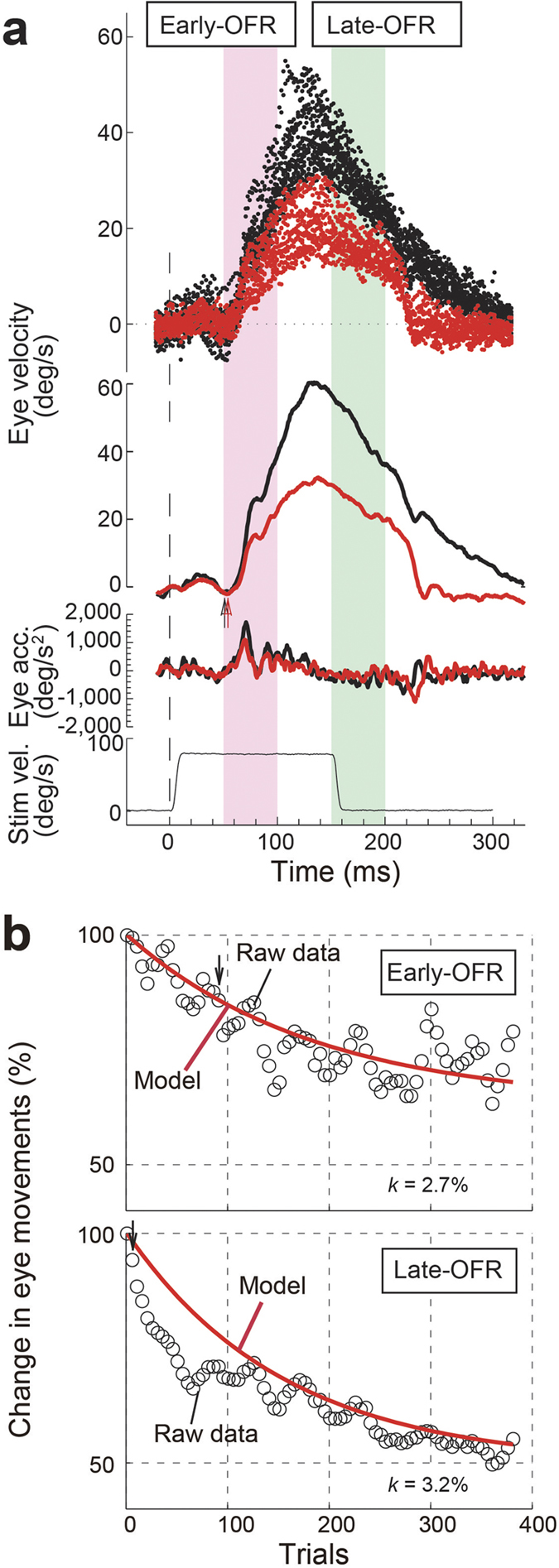
Sample response profiles from monkey U to the velocity step-down sequence paradigm. First ramps 80°/s downward; second ramps 0°/s. (**a**) Ocular response profiles. The ocular following responses (OFRs) during adaptation in the initial stage, trials 1–20, are shown in black, and those in the later stage, trials 381–400, are shown in red. From top to bottom, the graphs depict superimposed horizontal eye velocity profiles, average eye velocity, average eye acceleration (acc), and stimulus velocity (Stim vel) profiles. Arrows show the estimated time of ocular response onset, 51 ms for the initial stage of adaptation and 54 ms for the later stage. Upward deflections represent motion in the direction of the first ramp. To examine learning effects on the OFR, we quantitatively integrated the eye velocity over the time periods of 50–100 ms (magenta shaded column) and 150–200 ms (green shaded column), and calculated the changes in eye position for the early- and late-OFRs. (**b**) Learning effect curves of the OFRs. The moving averages of the OFRs over the time periods of 50–100 ms (early-OFRs) and 150–200 ms (late-OFRs) are shown (open circles). Arrows indicate the trial-windows when the responses started to change significantly. The red line in each panel indicates the fitted curve by the discrete model.

Figure 1a shows sample response profiles that illustrate the effects of the adaptive paradigm (velocity step-down sequences) on OFR velocity. Velocity step-down sequences resulted in clear decreases in tracking responses; the adapted profile began to deviate from its preadaptation approximately 70 ms after the first velocity-step onset and 80 ms before the second velocity-step onset, which was consistent with a previous report^7^. In other words, the decrease in the OFR gain reduced the retinal error during the second step (150 ms) but increased the retinal error during the first step (~80 ms). The latencies of the OFRs were similar during the course of the adaptation (51 ms for the initial stage, trials 1–20 and 54 ms for the later stage, trials 381–400).

To examine learning effects on the OFRs, we quantitatively integrated the eye velocity over the time periods 50–100 ms and 150–200 ms and calculated the changes in eye position for the early and late periods. The time courses of the learning effects on the OFRs (the early-OFR in the magenta shaded column and the late-OFR in the green shaded column in Fig. 1a) are shown in Fig. 1b. The OFRs gradually decreased in both the early and late periods. The decreases in the early- and late-OFRs reached a significant level between trials 96–115 and 6–25, respectively. To characterize the time course of the behavioral changes (400 trials), we used a discrete model^19^,^20^. In Fig. 1b, the thick red lines show the model predictions and indicate that the discrete model provided a good fit to the observed data. The correlation coefficients (*r*) between the change in eye movements and its summation were −0.85 and −0.94 in the early-OFRs and the late-OFRs, respectively (*p* < 0.0001 in both OFRs). The correction rate (*k*) was 2.7% for the early-OFRs and 3.2% for the late-OFRs.

The initial drastic adaptive gain reduction was induced by the first 100 adaptation stimuli in most instances. Therefore, to study the effects of learning on neuronal and ocular responses, we selected 55 neurons (55/58, 94.8%) whose responses were recorded for more than 120 trials during the learning session.

When we fit the discrete model to the behavioral changes in ocular responses, the correlations were significant in 42/55 (76.4%) and 49/55 (89.1%) of the early- and late-OFRs, respectively (*p* < 0.05). The *k* values were similarly distributed for the early- (mean ± standard deviation, −9.2 ± 4.6) and late-OFRs (−10.1 ± 4.4), and the difference between the distributions was not significant (*p* = 0.29, nonparametric Mann-Whitney U tests).

### The relationship between neuronal and ocular responses and retinal errors during learning

#### The initial learning stage

**Figure 2:**
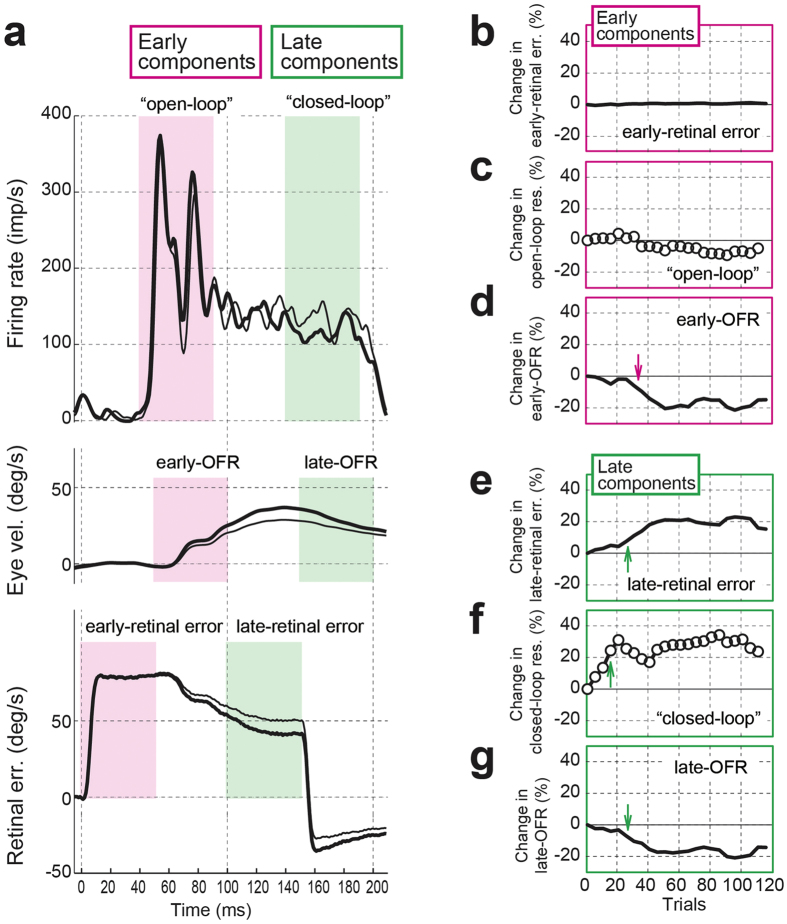
Effects of the velocity step-down paradigm on the neuronal/ocular responses. (**a**) From top to bottom, the graphs depict the superimposed discharge rate of a dorsal medial superior temporal (MSTd) neuron, eye velocity (vel), and retinal error (err) velocity profiles during the initial learning stage to the velocity step-down sequence paradigm of Monkey U. The preferred stimulus of the neuron was 80°/s upward. All profiles were aligned at the stimulus onset. The initial learning stage is divided into the starting trials, consisting of trials 1–40 (thick solid lines), and the ending trials, consisting of trials 81–120 (thin solid lines). Magenta shaded columns show the time periods used to analyze the relationship between neuronal and ocular responses. Green shaded columns show the time periods used to analyze the relationship between visual motion on the retina and neuronal responses. (**b–g**) Moving averages. The first set of 20 trials was used to normalize the remaining sets, and shown are the time courses of the changes in the early-retinal error (**b**), the “open-loop” responses of the neuron (**c**), the early-ocular following response (OFR) (**d**), the late-retinal error (**e**), the “closed-loop” responses of the neuron (**f**), and the late-OFR (**g**). The arrow in each panel (**d–g**) shows the time of significant change.

Figure 2a shows the mean change in neuronal and ocular response profiles and retinal error profiles during the initial learning stage (1–120 trials). In the initial stage of learning (trials 1–40 versus trials 81–120), the velocity step-down sequences immediately decreased the amplitudes of OFRs, which caused slight increases in retinal errors. The neuron did not show a clear change in its early component (“open-loop” response). However, in the late component (“closed-loop” response, green column in Fig. 2a), slight differences were observed between the responses in trials 1–40 (thick line) and those in trials 81–120 (thin line).

To quantitatively analyze the relationship between neuronal activity and sensory/motor signals, the mean firing rate was calculated in the two time periods (early components and late components). In the early components, the progress of adaptation made no difference in the causal retinal errors for the early-OFR (Fig. 2b). In addition, the neuronal responses (“open-loop” responses) showed no significant change (Fig. 2c) despite the significant decreases in the early-OFRs (Fig. 2d: from trials 36–55). In the late components, the causal retinal errors that preceded the late-OFR significantly increased as the adaptation progressed (Fig. 2e: from trials 26–45). The neuronal responses (“closed-loop” responses) significantly increased (trials 16–35, Fig. 2f), whereas the late-OFRs significantly decreased (trials 26–45, Fig. 2g).

**Figure 3:**
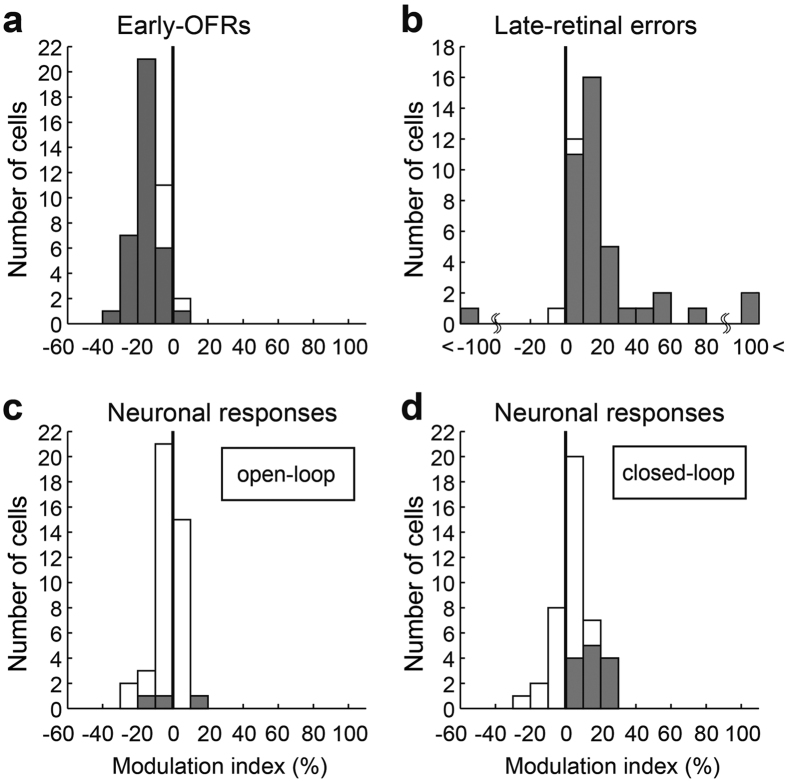
The distributions of the modulation indices of 42 MSTd neurons at the end of initial learning (trials 101–120). (**a**) The distribution of the modulation indices for the early-ocular following response (OFR). (**b**) The distribution of the modulation indices for the late-retinal error. (**c**) The distribution of the modulation indices for the neuronal responses during the “open-loop” period. (**d**) The distribution of the modulation indices for the neuronal responses during the “closed-loop” period. The shaded bars indicate the MSTd neurons showing significant changes.

To quantify the changes in the initial learning stage (~120 trials), we calculated modulation indices for the early-OFRs, the late-retinal errors, and the neuronal firing rate (“open-loop” and “closed-loop” responses), which was defined as follows:


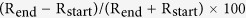


where R_start_ and R_end_ are the ocular responses, retinal errors, and neuronal responses at the start (trials 1–20) and end (trials 101–120) of the initial learning stage, respectively. The index was positive when the ocular responses, retinal errors, or neuronal responses increased from the first 20 trials of learning. Figure 3 shows these indices for the 42 learning sessions (42/55) for which the corresponding learning curves of the early-OFRs showed significant correlations (*p* < 0.05) with the discrete model. Most of the modulation indices of the early-OFRs and the late-retinal errors were distributed in the negative (Fig. 3a; −13.5 ± 7.6%) and positive (Fig. 3b; 23.01 ± 26.7%) areas, respectively. Thirty-six sessions (36/42) produced significant decreases in the early-OFRs, and 40 sessions (40/42) produced significant increases in late-retinal errors. For neuronal responses, the modulation indices of the “open-loop” and “closed-loop” responses tended to distribute in negative (Fig. 3c, −2.4 ± 8.2%) and positive (Fig. 3d, 4.9 ± 10.8%) areas, respectively. Only three MSTd neurons (3/42, 7.1%) significantly changed their “open-loop” responses; the discharges were decreased in 2 neurons (2/3) and increased in one neuron (1/3) (Fig. 3c). Thirteen MSTd neurons (13/42, 31.0%) exhibited significantly different “closed-loop” responses, and increased responses were observed in all 13 neurons (13/13) (Fig. 3d).

#### The later learning stage

After the later learning stage, all 34 recording sessions (more than 400 trials) produced learning curves of the early-OFRs with significant correlations (*p* < 0.05) to the discrete model (see Supplementary Table S1). In the later learning stage (trials 1–20 versus trials 381–400), all of the sessions produced significant decreases in the early-OFRs (−18.2 ± 14.3%) and significant increases in the late-retinal errors (25.9 ± 25.5%). The modulation indices of the “open-loop” and “closed-loop” responses of 34 MSTd neurons tended to distribute in negative and positive areas, respectively (−6.0 ± 10.2%, 6.3 ± 11.6%, respectively). Nine MSTd neurons (9/34, 26.5%) exhibited significantly different “open-loop” responses, and 8 of them (8/9) exhibited decreased responses. In addition, fourteen MSTd neurons (14/34, 41.2%) exhibited significantly different “closed-loop” responses, and 11 of them (11/14) exhibited increased responses.

#### The population average

**Figure 4:**
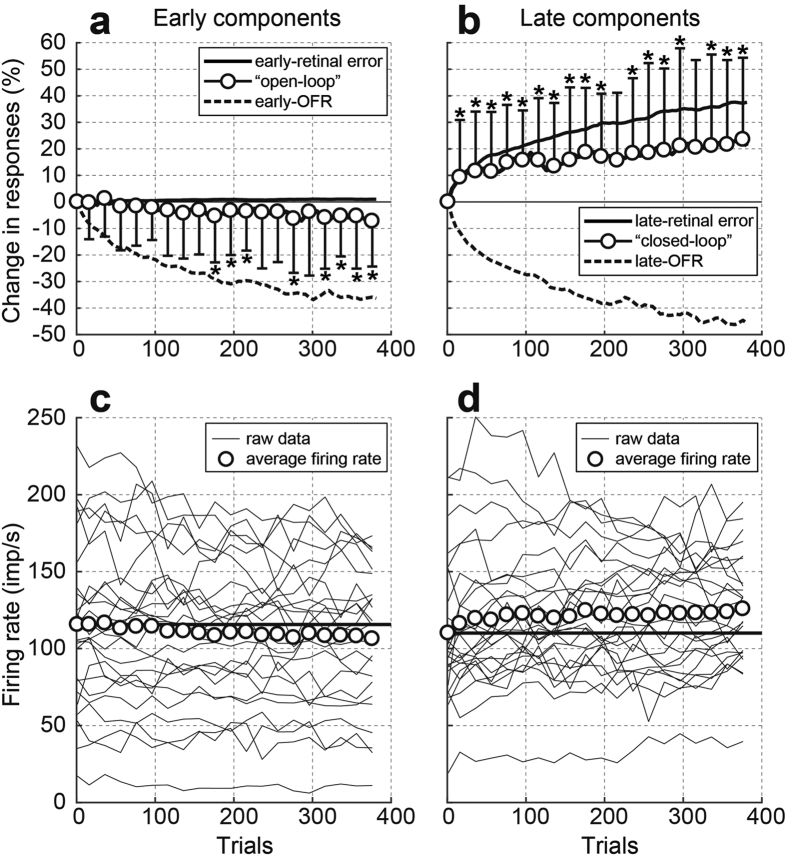
The relationship between the time courses of the neuronal population averages, normalized averages of the OFRs, and retinal errors. (**a,b**) The neuronal responses are plotted by open circles and thick lines (mean ± standard deviation). The ocular responses and retinal errors are plotted by dashed and solid lines, respectively. (**a**) The normalized average of the early-retinal errors (0–50 ms), the neuronal population average of the “open-loop” responses (40–90 ms), and the normalized average of the early-ocular following responses (OFRs) (50–100 ms) are depicted. (**b**) Shown are the normalized average of the late-retinal errors (100–150 ms), the neuronal population average of the “closed-loop” responses (140–190 ms), and the normalized average of the late-OFRs (150–200 ms). (**c**) Raw firing rate of each MSTd neuron (thin lines) and the average firing rate of the neurons (open circles) during the “open-loop” period. (**d**) Raw firing rate of each MSTd neuron and the average firing rate of the neurons during the “closed-loop” period. Note: In (**a**,**b**), to calculate the neuronal population average the firing rate of each neuron was normalized with the first set of 20 trials. In (**c**,**d**) the firing rates of the neurons were not normalized to calculate the average firing rate.

To study the effects of adaptive motor learning from the initial stage to the later stage over a longer time course, we selected 25 neurons (25/34, 73.5%) with learning curves of the early-OFRs that showed significant correlations (*p* < 0.05) with the discrete model (*p* < 0.05, |*r*| > 0.6). Figure 4a,b show the relationships between the time courses of the neuronal population averages (mean of all of the normalized neuronal responses, open circles), the normalized averages of the OFRs (broken line), and retinal errors (continuous line) for the early (a) and late (b) components, respectively. The early- and late-OFRs decreased immediately following the velocity step-down sequences (Fig. 4a,b). In contrast, the early-retinal errors did not change (Fig. 4a), but the late-retinal errors increased due to the decrease in eye movements (late-OFR) as learning progressed (Fig. 4b). Regarding the early components, the neuronal population average (“open-loop” responses) did not change significantly during the initial learning stage (trials 21–180). In trials 41–100, the neuronal population average (“open-loop” responses) decreased only by 0.9% from the first trials (1–20), but the normalized average of the early-OFR was decreased by 17.5%. In contrast, the neuronal population average (“open-loop” responses) decreased significantly but modestly in the later learning stage (trials 181–400 in Fig. 4a). In trials 341–400, the neuronal population average (“open-loop” responses) decreased by 6.2%, and the normalized average of the early-OFR decreased by 36.3%. Although these modest changes were in the same direction as those of the ocular responses, the time courses of their changes were different. In other words, the changes in the early-OFR were apparent from the start of the initial learning stage but were not observed in the neuronal population average (“open-loop” responses). Regarding the late components, the neuronal population average (“closed-loop” responses) showed an immediate significant increase and continued to increase throughout the learning (Fig. 4b), as did the number of late-retinal errors. In trials 41–100, the neuronal population average (“closed-loop” responses) increased by 13.0% from the first trials (1–20). The normalized average of the late-retinal error increased by 17.5%. In addition, in trials 341–400, the neuronal population average (“closed-loop” responses) increased by 21.8%, and the late-retinal error increased by 36.9%.

To understand the contribution of individual neurons from another aspect, we also calculated the magnitudes of raw firing rate without normalizing the responses. Figure 4c and d show the time courses of the “open-loop” and “closed-loop” responses, respectively. Both time courses were similar to those of the neuronal population averages in Fig. 4a,b, which confirmed the results.

### Reconstruction of the temporal firing pattern

We also examined the relationship between neuronal activity and retinal errors to determine the type of visual information (acceleration and/or velocity of retinal errors) that is encoded in the firing patterns of the MSTd neurons. For this, we analyzed temporal firing patterns (0–190 ms from the first velocity-step onset) using a second-order linear-regression model^21^.

**Figure 5:**
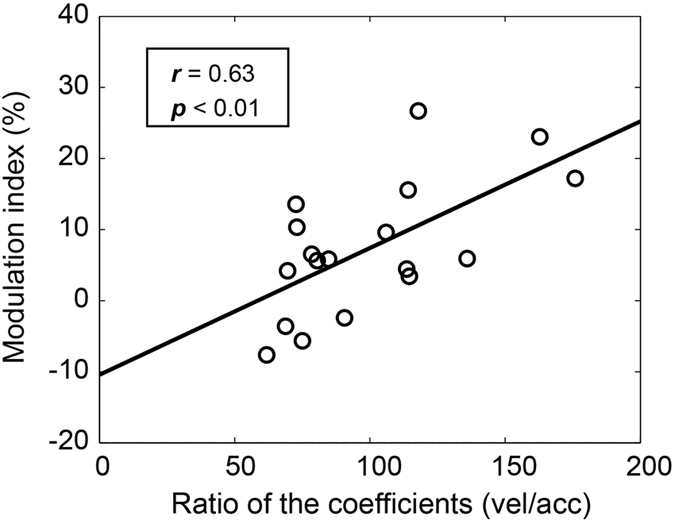
The relationship between the ratio of coefficient [velocity (vel)/acceleration (acc)] and modulation index. The modulation index in the initial stage of motor learning (trials 101–120) plotted against the ratio of coefficients (vel/acc) for 18 MSTd neurons and the regression line.

The retinal error model showed a good fit to the data in most cases (R^2^ > 0.7 in 29/34 MSTd neurons, 85.3%), which was consistent with our previous report^21^,^22^. The distribution of the coefficients of determination from the retinal error model was significantly better than that of the eye movement model (*p* < 0.05, nonparametric Mann-Whitney U tests). This finding suggests that information on retinal errors is represented in the temporal firing patterns of the MSTd neurons. Furthermore, we selected 18 MSTd neurons that could be reconstructed with the model with reliable parameters (R^2^ > 0.7 and *p* < 0.05), and calculated the ratios of their acceleration and velocity coefficients. The ratios were widely distributed (99.8 ± 33.1), which demonstrated that there were various cell types in the MSTd area. Moreover, we examined whether different cell types had different changes in neuronal responses during learning. As shown in Fig. 5, neurons with relatively larger ratios had greater modulation indices (*r* = 0.63, *p* < 0.01), which suggested that the velocity-dominant cells tended to increase their “closed-loop” responses during learning. However, the velocity/acceleration ratio did not show a significant correlation with the modulation indices of the “open-loop” responses (*r* = 0.38, *p* = 0.12).

## Discussion

The goal of the present study was to examine the neural basis of adaptive motor learning in the reflexive system (OFR), which underlies the execution of appropriate motor behaviors. The double velocity-step sequence paradigm has been shown to induce adaptive gain modulation of slow tracking eye movements, such as the OFR^7^ and smooth pursuit^23^,^24^, and thus has been used to study the neuronal basis of adaptive motor learning in the visual system. In the current study, a double velocity-step paradigm was adopted to induce the adaptive gain modulation of the OFR. In this paradigm, the initial step (0–150 ms from the stimulus onset) elicited the OFR, and the subsequent change in the stimulus velocity from the second step (150–300 ms from the stimulus onset, 0°/s) yielded sudden large retinal errors, which facilitated the adaptive gain down-modulation (motor-learning process) of the ocular responses. Through repeated exposure to the step-down sequences, we confirmed that the OFRs decreased approximately 70 ms after the first velocity-step and 80 ms before the second velocity-step. As a result, the retinal errors during the second-step decreased as the adaptation progressed, whereas the retinal errors during the first-step increased (the late-retinal errors, measured 100–150 ms from the first velocity-step onset). These findings are consistent with the results of the original study of the speed-step paradigms by Miles and Kawano^7^, who found that the latency of the earliest changes of the OFRs ranged from ~60 to ~110 ms, earlier than the second step onset. They suggested that the adaptive mechanism mainly uses the retinal error during the second velocity-step (150–300 ms period) to modulate the OFR gain because the repeated exposures to 150-ms ramps (blanking the screen after a 150 ms constant velocity stimulus) did not induce an adaptive modulation of the OFRs. Because the duration of the first step was 150 ms throughout the present study, the detailed relationship between the latency of the adaptive changes and the second step onset is unknown^7^. Notably, despite the similar findings of the two studies, there were methodological differences. In the previous study^7^, the experiment lasted for three days, whereas the current adaptation experiment (one session) lasted for approximately 25 minutes to ensure that the recording of the isolated MSTd neurons was maintained. Despite these methodological differences, repeated exposure to velocity step-down sequences resulted in considerable modulation of the OFRs, which suggests that appreciable changes are evident after only 100 trials. Furthermore, we found that the time course of motor learning can be interpreted with a discrete model. The distributions of the “correction rate,” *k*, in this study were similar to those in prism adaptation tasks that use reaching^25^,^26^.

During the course of the adaptive gain modulation of the OFR, we recorded the activity of the MSTd neurons. In the initial stage of adaptive motor learning (trials 1–100), we found differences between the neuronal responses during the “open-loop” and “closed-loop” periods. At the end of the initial learning stage (trials 101–120), the “open-loop” responses of the majority of the neurons (39/42, 92.9%) had not significantly changed, but the responses of a few neurons (3/42, 7.1%) had significantly changed. In contrast, approximately one-third of the MSTd neurons (13/42, 31.0%) exhibited significantly higher “closed-loop” responses in accordance with an increase in retinal error velocity (late-retinal error), although no significant changes were observed in the remaining neurons. Consistent with the results of individual neurons, the neuronal population average of the “closed-loop” responses was significantly higher (13.0%), in contrast to the average of the “open-loop” responses, which did not change significantly (0.9%).

In the later stage of adaptive motor learning (trials 381–400), the “open-loop” responses changed significantly in approximately one quarter of the MSTd neurons (9/34, 26.5%), as did the “closed-loop” responses in approximately 40% of the MSTd neurons (14/34, 41.2%). As shown in Fig. 4a, after the monkeys had experienced 340 velocity step-down sequences (trials 341–400), the normalized average of the early-OFR was reduced by 36.3% (trials 1–20). However, the decrease in the neuronal population average of the “open-loop” responses was only 6.2%, which is 17.2% of the change in the early-OFR. Thus, the changes in MSTd firing were not sufficiently substantial to fully explain the reduction of the OFRs, although the comparison between behavior and neuronal firing might be overly simplistic. Although, the increase in the “closed-loop” responses of the MSTd was 59.1% of the change in the normalized average of the later-retinal error (21.8%/36.9%, Fig. 4b).

The linear-regression analysis showed a good reconstruction of the neuronal responses by the retinal error model, which supports the idea that these neurons encode retinal errors. This analysis also confirmed the findings of a previous study^21^ that indicated that there are MSTd neurons with different dominances for encoding retinal error, velocity, and/or acceleration. Furthermore, the correlation analyses revealed that MSTd neurons with greater velocity-sensitivity were more influenced by changes in retinal errors during motor learning.

Based on previous single-unit recording studies and chemical lesion experiments that addressed OFRs^22^,^27^,^28^,^29^, Takemura and Kawano^22^ suggested an information processing stream that regulates the OFR. Briefly, individual MST and DLPN neurons encode selective aspects of sensory (retinal) information that is related to the motion in a visual scene and provide this information to the VPFL of the cerebellum. Next, Purkinje cells (P-cells) in the VPFL encode the dynamic command signals for the associated motor response of the OFR; the sensory-to-motor transformation for the OFR occurs in the P-cells in the VPFL of the cerebellum. The cerebellum has long been considered a primary site of motor learning; this is a theory that is supported by other studies of motor learning, as in classical eyelid conditioning, vestibular ocular reflexes, and smooth pursuit eye movements^30^,^31^,^32^,^33^,^34^,^35^,^36^. Regarding smooth pursuit eye movements, the MST area has also been suggested to play a significant role in motor learning by providing an input signal to the cerebellum^37^,^38^.

As described above, in the initial learning stage, most MSTd neurons (39/42, 92.9%) did not exhibit significantly different “open-loop” responses, which did not correspond with the modulation of the OFR gain. Furthermore, the time-course of the neuronal population average did not show such a steep incline as observed in the early-OFR (20–60 trials in Fig. 4a). These results suggested that the MST area was neither the site of nor a site that was downstream of the adaptive gain modulation in the initial learning stage. To adaptively modulate the OFRs in the initial learning stage, the neuronal responses that occur downstream of the MST area or in alternative pathways (e.g., the nucleus of the optic tract) should be modulated. Although we found a subset of the MSTd neurons with significant changes in “open-loop” responses in the later learning stage, it is premature to conclude that the change in the firing rates of such neurons during the later learning stage is the cause or consequence of motor learning.

The MSTd neurons in this study all responded to planar motion. Previous studies have indicated that such neurons in the MST area also contain information on vergence angle^13^, binocular disparity^14^, and post-saccadic delay^15^ in their short-latency discharges. Therefore, these MST neurons may be involved in the contextual gain modulation of the OFR using the vergence angle^13^, binocular disparity^14^, and post-saccadic delay^15^,^16^ information. In other words, the MST area is considered to be the site of, or downstream of, the contextual gain modulation of the OFR that is associated with these three factors. In contrast, the present findings suggest that the MSTd area might be upstream of the adaptive gain modulation (motor learning) of the OFR, and provide retinal error information during adaptive learning. In other words, the adaptive gain modulation (motor learning) system for the OFR may function through a different mechanism compared to the contextual gain modulation system.

As described above, approximately one-third of MSTd neurons (13/42, 31.0%) exhibited significantly different “closed-loop” responses in the initial stage of motor learning, and such significant changes were observed throughout the course of the adaptation. The time-course of the neuronal population average was similar to that of the retinal error average (Fig. 4b). These results are consistent with the idea that the MST area provides an input signal (retinal error) to the VPFL of the cerebellum via the DLPN to elicit the OFR. We assume that the MST area encodes causal retinal errors for motor learning in the neuronal activities, and provides the information to the cerebellum continuously because the cerebellum is suggested to be the site of the motor learning of the OFR, based on other motor learning studies. However, in this paper, we could not obtain adequate information about the modulation of the neuronal activities that possibly encoded the retinal errors during the second velocity-step period (causal retinal errors for motor learning) because the neuronal responses to the retinal error stimuli in the anti-preferred directions were almost suppressed throughout the adaptation trials. Further study of the responses of neurons whose preferred directions were opposite to the first velocity-step of the adaptation paradigm would clarify the role of the MSTd neurons in encoding the causal retinal error information for motor learning.

The results of the present study have revealed that the MSTd area may provide visual error information to the downstream structures during adaptive modulation of the OFR. However, because the recording site of this study was limited to the MSTd area, the results are not sufficient to completely delineate its neural mechanism. Additional studies in which neuronal activity is recorded on a trial-by-trial basis during motor learning in the cerebellum and other downstream structures, including the pontine nucleus and nucleus of the optic tract, would help to fully elucidate the core mechanisms involved in motor learning of the OFR.

## Methods

Data were collected from three adolescent monkeys (*Macaca fuscata*) that weighed 7.7–8.4 kg. All of the experimental protocols were approved by the Animal Care and Use Committee of the National Institute of Advanced Industrial Science and Technology, which complied with the guidelines established in the Public Health Service Guide for the Care and Use of Laboratory Animals (Approval number: 32–06–011, Dou2011-050, Dou2012-050, Dou2013-050, Dou2014-050, Dou2015-050, Dou2016-050). Most of the methods employed have been described previously^7^,^12^.

### Animal preparation

All three animals were previously trained to fixate on a small target to obtain a liquid reward^39^. Under aseptic conditions and pentobarbital sodium anesthesia with non-steroidal anti-inflammatory drugs, each monkey was fitted with a head holder to allow the head to be fixed in a standard stereotaxic position during the experiments. The monkeys were also fitted with cylinders for chronic recording of single neuron activity in the MST area. The scleral search coils that were used to measure eye movements were implanted in both eyes according to the technique of Judge, *et al*.^40^.

### Behavioral paradigm

During the recording sessions, the monkey sat in a primate chair with its head secured by a head holder. The animal faced a large (114 × 114 cm) translucent tangent screen (viewing distance: 50 cm; subtending: 90° × 90°). The visual image was back-projected onto the screen as a random-dot pattern. The density of dots was 50%. The smallest dots in the pattern subtended ~1.5° of arc, and the luminance ranged from 5.4 cd/m^2^ (white dots) to 0.6 cd/m^2^ (black background areas). Mirror galvanometers in an *X*/*Y* configuration in the projection path allowed the horizontal and vertical positions of the pattern to be controlled.

A velocity step-down sequence paradigm was used in this study (Fig. 1). At the start of each double-step sequence, the first movement of the random dot pattern lasted 150 ms at a constant velocity in one direction and started 50 ms after the end of the centering saccade. After 150 ms, the drift velocity abruptly changed (a decrease to 0°/s). This stationary random dot pattern (0°/s) was presented for 150 ms to impose an abnormal retinal slip. The screen was then blank for 500–2,000 ms. This blank screen deprived the OFR system of further visual feedback until the eyes had returned to rest. Following the appearance of the blank screen, the pattern was reset to its initial start position and was ready for the next trial. To help the monkey remain alert, it was given a drop of fruit juice at the end of each trial.

### Single-cell recording technique

A hydraulic microdrive (Narishige MO-9, Tokyo, Japan) was mounted onto the recording cylinder, and glass-coated tungsten microelectrodes were used for the initial identification and mapping of MST and medial temporal (MT) cortical areas. Subsequently, a fixed grid system^41^ was used to introduce flexible tungsten electrodes into the MSTd area for single-cell recordings. The tips of the guide tubes were positioned 3–4 mm above the MSTd area.

### Selection of neurons

Our primary aim was to investigate the responses of OFR-related neurons in the MSTd area during motor learning. To achieve this, we initially selected neurons based on the sensitivity of their discharge to the moving visual scene that elicited OFRs. After isolating a single neuron, we observed its responses to the moving visual scene at 80°/s in eight directions. Further studies were performed specifically with neurons that showed directional selectivity. After determining the preferred direction of the neuron, we observed its responses to the moving visual scene in the preferred direction at five speeds ranging from 10, 20, 40, 80, and 160°/s. Most of the direction-selective MSTd neurons showed the strongest responses at high stimulus speeds (80 or 160°/s). Finally, we applied a velocity step-down sequence in which the first velocity step was optimal for the activation of the isolated neuron (in terms of speed and direction). The monkey performed 200–2,000 trials of velocity step-down sequences while we isolated a single neuron and recorded the ocular and neuronal responses.

### Data collection and assessment of learning effects on the OFR

The eye-position measures were hardware differentiated to obtain eye velocity measures. These position and velocity voltage signals were smoothed with a 6-pole Bessel filter (3 dB at 100 Hz) and then digitized at 500 Hz with a resolution of 12 bits. Voltage signals that encoded the positions of the mirror galvanometers that controlled the positions of the patterns and target spots on the screen were sampled at 250 Hz with a resolution of 12 bits. Neuronal activity was recorded via standard extracellular recording techniques. A time-amplitude window discriminator was used to obtain spike occurrences at 1 kHz. Trials with saccadic intrusions during the experiment were deleted using an interactive computer program created in MATLAB (MathWorks, Natick, MA, USA).

Eye velocity data were averaged to obtain a temporal profile. The average eye velocity profiles were low-pass filtered (Butterworth filter, −3 dB at 50 Hz) and differentiated using a digital filter to yield mean eye acceleration profiles. The latencies of the OFRs were determined by eye accelerations first exceeding 100°/s^2^.

To quantitatively analyze the OFR, we calculated the mean change in eye position over a time period of 50–100 ms from the stimulus onset, termed the “early-OFR,” which corresponded to the open-loop period of the OFR; the early-OFR occurred before eye movements had any chance to affect the visual stimuli on the retina because of the OFRs’ latency (~50 ms). The mean change in eye position over a time period of 150–200 ms was also calculated and termed the “late-OFR”. We applied a trial-window for 20 trials that moved in 5 trial steps to calculate the time course of changes in ocular responses during velocity step-down sequences (120 trials) in each session. The learning effects were quantified using a discrete model^19^ formulated as follows:


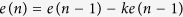


where *e(n)* indicates change in eye position (%) in the *n*th trial window. The error was plotted against the summation of errors to test if there was a linear correlation between them. The slope of the regression line gave an estimate of *k*, which indicated a rate constant of decay (‘correction rate’). To evaluate the performance of the model, we used the *p*-value and the correlation coefficient (*r*).

### Analysis

#### Assessment of neuronal activity in the MSTd area

To study the effects of learning, the temporal profiles of neuronal and ocular responses from 20 trials were averaged for a given adaptation stage in each cell after trials with saccadic intrusion were excluded. Subsequently, the responses were low-pass filtered (Butterworth filter, −3 dB at 50 Hz). The temporal profiles of retinal errors as a visual motion signal were obtained by subtracting eye movements from mirror movements. Furthermore, we also focused on two time periods that were related to neuronal activity in the MSTd area—early and late. For the early components, we measured the “open-loop” responses of a given neuron (40–90 ms), the early-OFR (50–100 ms), and the mean change in retinal error over a time period of 0–50 ms (the early-retinal error). For the late components, we measured the “closed-loop” responses of a given neuron (140–190 ms), the late-OFR (150–200 ms), and the mean change in retinal error over a time period of 100–150 ms (the late-retinal error). There was a 10-ms difference in the start of the time intervals over which we measured the ocular and neuronal responses because the OFR-related activity of the neurons in the MST area has been shown to precede the associated eye movements by approximately 10 ms (Kawano *et al*., 1994). To examine the time courses of changes in neuronal and ocular responses and retinal errors during velocity step-down sequences in each session, we applied a trial-window for 20 trials that moved in 5 trial steps. We used nonparametric Mann-Whitney U tests to compare the values obtained in each trial-window with the first trial-window. Next, we identified the first five consecutive trial-windows that were significantly different from the first trial-window (p < 0.05). We defined the first 5 significantly different consecutive trial-windows as the trial-window that started with a significant change in neuronal and ocular responses and retinal errors. To show the learning curve for these responses, the average in each trial-window was normalized with the data of the first 20 trials.

#### A linear-regression model of the firing rate

To quantitatively analyze the contribution of sensory information to neural activity, we applied a regression analysis to the neuronal activity data (Takemura *et al*. 2001). The equation that was used for this analysis is as follows:


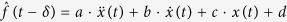


where 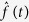, 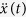, 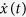, 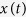, and δ are the reconstructed firing frequency of a neuron, the acceleration, velocity, and position of retinal errors at time *t*, and the time delay, respectively. Four coefficients (*a, b, c, and d)* and the time delay (δ) were estimated to minimize the squared estimation error. To estimate each coefficient at a particular δ, a linear-regression retinal error model was applied to the firing pattern from the stimulus onset (the first step onset) to 40 ms after the end of the first step; the duration was 190 ms. The search range for δ was limited to −80 to −20 ms. To evaluate the performance of the model, we used the coefficient of determination (R^2^).

### Histology

After recordings were completed with the three monkeys, two were deeply anesthetized with pentobarbital and perfused through the heart with saline, followed by 10% formalin. The third monkey was not sacrificed as it was being used for other experiments. The animals’ brains were removed, and frozen sections were cut at 50 μm in the sagittal plane, mounted onto microscope slides, and stained with cresyl violet and a modified silver stain for cell bodies and myelinated fibers, respectively. Recording sites were verified using both the Nissl method and a myelin silver stain method. The superior temporal sulcus and surrounding cortex were displayed on unfolded maps according to the method of Van Essen and Maunsell^42^. Electrolytic lesions facilitated histological reconstruction of the electrode tracks, and the recording sites were verified using both Nissl stain and myelination. Electrode position in the MSTd area was verified using magnetic resonance imaging and physiological characteristics of the MSTd area, including direction selectivity and receptive field size.

## Additional Information

**How to cite this article:** Takemura, A. *et al*. Neural activity in the dorsal medial superior temporal area of monkeys represents retinal error during adaptive motor learning. *Sci. Rep.* **7**, 40939; doi: 10.1038/srep40939 (2017).

**Publisher's note:** Springer Nature remains neutral with regard to jurisdictional claims in published maps and institutional affiliations.

## Supplementary Material

Supplementary Table S1
