# Supplementary material for: Neural activity in the dorsal medial superior temporal area of monkeys represents retinal error during adaptive motor learning
Source: Sci Rep. 2017 Jan 19;7:40939. doi: 10.1038/srep40939 (PMC5244411; doi:10.1038/srep40939)
Supplement: Supplementary Table S1 [file srep40939-s1.pdf]

Title: Neural activity in the dorsal medial superior temporal area of monkeys represents retinal error during adaptive motor learning

Authors: Aya Takemura[1,*], Tomoyo Ofuji[1], Kenichiro Miura[2], and Kenji Kawano[2]

Affiliations:

[1]Human Informatics Research Institute, National Institute of Advanced Industrial Science and Technology, Ibaraki 305-8568, Japan

[2]Department of Integrative Brain Science, Graduate School of Medicine, Kyoto University, Kyoto, 606-8501, Japan

[*]**Corresponding author:** Aya Takemura

Human Informatics Research Institute, AIST, Tsukuba Central 2,

1-1-1, Umezono, Tsukuba, Ibaraki 305-8568, Japan

Phone: +81-29-861-3426

Fax: +81-29-861-5849

E-mail: a.takemura@aist.go.jp

Monkey	Side of MST	# of trials	Pref dir.	Pref spd	MI (120)			Statistics (120)			MI (400)			Statistics (400)		
					OFR	open-loop	closed-loop	*p*	*k*	*r*	OFR	open-loop	closed-loop	*p*	*K*	*r*
s	L	81	L	80	N/A	N/A	N/A	N/A	N/A	N/A	N/A	N/A	N/A	N/A	N/A	N/A
s	L	214	L	80	-18.8	18.7	23.1	0.00	-17.8	-0.84	N/A	N/A	N/A	N/A	N/A	N/A
s	L	416	R	80	-14.5	3.6	17.2	0.00	-10.6	-0.72	-20.5	-14.5	11.8	0.00	-2.7	-0.78
s	L	494	L	80	-17.6	1.0	4.5	0.00	-6.4	-0.78	-28.4	0.3	13.8	0.00	-2.6	-0.71
s	L	500	D	80	-19.7	-8.8	-3.6	0.00	-20.3	-0.92	-30.0	-10.7	1.8	0.00	-4.1	-0.92
s	L	500	D	80	-16.1	-3.9	3.6	0.00	-11.5	-0.71	-27.7	-9.6	1.0	0.00	-3.7	-0.95
s	L	500	R	80	-7.6	-21.2	15.6	0.69	-0.9	-0.10	-24.1	-27.6	33.0	0.00	-2.2	-0.75
s	L	500	R	80	0.1	-3.4	-3.5	0.41	2.0	0.20	-19.0	-9.2	1.6	0.02	-0.8	-0.27
s	L	999	R	80	-3.3	-0.1	-3.0	0.00	-10.6	-0.63	-14.0	-3.3	1.7	0.00	-3.5	-0.8
s	R	397	R	80	-14.8	-5.5	3.4	0.00	-11.7	-0.92	N/A	N/A	N/A	N/A	N/A	N/A
u	L	38	L	160	N/A	N/A	N/A	N/A	N/A	N/A	N/A	N/A	N/A	N/A	N/A	N/A
u	L	123	L	80	-13.3	-9.3	-13.6	0.00	-7.8	-0.90	N/A	N/A	N/A	N/A	N/A	N/A
u	L	175	R	80	-24.2	-4.3	3.5	0.00	-12.9	-0.92	N/A	N/A	N/A	N/A	N/A	N/A
u	L	218	D	80	-10.0	-25.6	-17.9	0.00	-13.0	-0.93	N/A	N/A	N/A	N/A	N/A	N/A
u	L	243	L	80	-4.8	0.1	-6.1	0.02	-4.6	-0.52	N/A	N/A	N/A	N/A	N/A	N/A
u	L	298	U	160	-7.8	12.9	-6.4	0.89	0.3	0.03	N/A	N/A	N/A	N/A	N/A	N/A
u	L	348	D	160	-10.0	-1.3	9.7	0.00	-5.9	-0.78	N/A	N/A	N/A	N/A	N/A	N/A
u	L	394	D	80	-3.1	-12.3	-6.8	0.09	-4.5	-0.39	N/A	N/A	N/A	N/A	N/A	N/A
u	L	500	R	80	-16.5	-5.3	-9.9	0.00	-13.0	-0.88	-27.9	-9.5	-6.3	0.00	-3.7	-0.89
u	L	600	L	160	3.1	-9.7	-7.6	0.30	1.6	0.24	-9.1	-1.4	-7.6	0.00	-1.3	-0.51
u	L	772	D	160	-11.8	-4.3	8.0	0.00	-9.7	-0.96	-21.1	-2.8	8.0	0.00	-3.7	-0.94
u	L	911	U	160	-11.8	-1.7	1.2	0.03	-6.5	-0.48	-19.0	-13.8	5.9	0.00	-2.1	-0.85
u	L	986	L	160	-10.6	-3.8	2.7	0.00	-11.6	-0.86	-39.0	-3.3	4.2	0.00	-2.0	-0.93
u	L	1000	D	80	-7.8	2.9	4.4	0.00	-6.3	-0.90	-11.3	5.5	-1.0	0.00	-3.3	-0.88
u	L	1000	U	80	-13.5	-4.3	5.6	0.00	-13.8	-0.94	-14.9	-7.5	5.3	0.01	-0.8	-0.28
u	L	1030	D	160	0.8	5.6	3.4	0.03	2.7	0.48	-14.0	8.5	10.6	0.00	-1.7	-0.55
u	L	1200	L	40	-11.3	8.7	26.7	0.00	-6.4	-0.81	-11.7	5.1	31.8	0.00	-2.6	-0.84
u	L	1773	U	160	-11.1	5.4	10.3	0.00	-5.7	-0.65	-29.4	-5.7	8.8	0.00	-2.6	-0.83
u	L	1962	U	80	-5.8	0.3	13.2	0.07	-3.6	-0.42	-15.2	-1.2	16.8	0.00	-3.3	-0.87
u	L	2000	U	80	-11.0	-3.5	13.6	0.00	-12.8	-0.87	-10.9	-9.4	9.4	0.00	-3.8	-0.82
u	R	48	D	80	N/A	N/A	N/A	N/A	N/A	N/A	N/A	N/A	N/A	N/A	N/A	N/A
u	R	145	R	20	-30.1	2.1	14.8	0.00	-10.4	-0.96	N/A	N/A	N/A	N/A	N/A	N/A
u	R	200	U	10	-8.3	21.5	47.9	0.75	-0.6	-0.08	N/A	N/A	N/A	N/A	N/A	N/A
u	R	200	U	10	-8.7	3.7	15.3	0.00	-5.5	-0.72	N/A	N/A	N/A	N/A	N/A	N/A
u	R	200	D	80	-16.1	2.0	22.6	0.01	-5.7	-0.58	N/A	N/A	N/A	N/A	N/A	N/A
u	R	200	L	80	-3.4	-46.2	-37.7	0.87	0.3	0.04	N/A	N/A	N/A	N/A	N/A	N/A
u	R	201	L	80	-12.6	-8.2	2.0	0.00	-7.5	-0.78	N/A	N/A	N/A	N/A	N/A	N/A
u	R	212	D	80	6.6	-3.1	-24.2	0.03	3.9	0.48	N/A	N/A	N/A	N/A	N/A	N/A
u	R	225	U	80	-19.8	-10.6	-9.3	0.00	-6.9	-0.84	N/A	N/A	N/A	N/A	N/A	N/A
u	R	241	U	40	-8.9	-22.9	-1.6	0.00	-6.6	-0.78	N/A	N/A	N/A	N/A	N/A	N/A
u	R	289	D	40	-8.5	2.4	8.3	0.00	-6.0	-0.68	N/A	N/A	N/A	N/A	N/A	N/A
u	R	377	R	80	-8.6	6.6	7.2	0.08	-3.5	-0.40	N/A	N/A	N/A	N/A	N/A	N/A
u	R	399	D	80	-5.3	-1.9	16.6	0.00	-6.4	-0.65	N/A	N/A	N/A	N/A	N/A	N/A
u	R	483	L	40	-24.8	-9.2	4.2	0.00	-11.5	-0.96	-25.4	-18.2	-0.8	0.00	-2.9	-0.53
u	R	500	R	20	14.0	1.2	-5.7	0.06	2.3	0.43	29.8	-3.5	-15.9	0.00	5.8	0.34
u	R	500	L	40	-21.6	-0.4	5.8	0.00	-9.6	-0.77	-25.5	-14.3	-9.1	0.00	-4.7	-0.88
u	R	500	D	80	-24.4	7.0	5.7	0.00	-12.4	-0.97	-43.9	5.1	18.3	0.00	-4.6	-0.98
u	R	500	D	80	-11.3	1.7	23.0	0.00	-8.7	-0.84	-22.9	17.4	28.4	0.00	-0.9	-0.68
u	R	500	D	80	-9.9	3.2	5.9	0.00	-8.8	-0.87	-27.4	2.1	9.8	0.00	-2.5	-0.96
u	R	952	L	160	-22.1	-8.8	-2.4	0.00	-10.1	-0.86	-33.2	-23.7	-19.0	0.00	-3.2	-0.97
u	R	995	R	20	-7.7	-2.9	-13.1	0.89	0.3	0.03	1.9	4.5	3.6	0.00	1.6	0.51
u	R	1489	D	20	-24.8	-10.3	5.1	0.00	-14.5	-0.98	-30.6	-6.1	6.8	0.00	-6.7	-0.77
u	R	1500	L	20	-24.4	-2.2	9.6	0.00	-15.2	-0.87	-10.9	-24.2	4.4	0.03	-0.8	-0.26
u	R	1500	D	40	-6.4	-5.7	6.5	0.50	-1.3	-0.16	-9.3	-3.0	12.7	0.00	-2.3	-0.77
u	R	2000	D	20	-4.9	-1.2	13.3	0.00	-7.9	-0.74	-14.6	0.5	10.6	0.00	-3.3	-0.88
u	R	2000	L	80	-16.9	9.7	-3.2	0.00	-13.3	-0.80	-25.5	5.5	-4.4	0.00	-1.9	-0.63
v	L	500	R	160	-6.3	-17.7	7.1	0.00	-7.7	-0.77	-9.1	-14.8	2.4	0.00	-5.0	-0.8
v	L	1000	U	80	7.4	-42.3	9.1	0.68	-1.0	-0.10	16.7	-21.0	16.5	0.00	4.2	0.51

Supplementary Table S1

We recorded the responses of 58 direction-selective neurons from the dorsal part of the MST (MSTd) to the adaptation stimulus in five hemispheres (Side of MST) of three monkeys (Monkey). The MSTd neurons in the present study showed a preferred direction (Pref dir.) and speed (Pref spd). This table shows the modulation indices (MI) for the early-OFRs (OFR), the neuronal firing rate (“open-loop” and “closed-loop” responses), and the statistics of the early-OFRs by the discrete model for the initial learning stage (120, i.e., trials 101-120) and the later learning stage (400, i.e., trials 381-400).
